# Temporal Rewiring of Innate Immunity by Vector-Borne Viruses for Host-Directed Antiviral Therapy

**DOI:** 10.3390/ijms27167292

**Published:** 2026-08-15

**Authors:** Eunji Kim, Andreas S. Baur, Jung-Hyun Lee

**Affiliations:** 1 Department of Life Science, University of Seoul, Seoul 02504, Republic of Korea; kimej0212@uos.ac.kr; 2 Department of Dermatology, Universitätsklinikum Erlangen, Friedrich-Alexander-University Erlangen-Nürnberg, 91054 Erlangen, Germany; andreas.baur@uk-erlangen.de

**Keywords:** vector-borne virus, innate immune response, interferon signaling, inflammatory pathology, immune timing, host-directed antiviral therapy

## Abstract

The geographic range and outbreak intensity of vector-borne viral infections are increasing as climate, land use, urbanization, and human mobility reshape vector ecology and human exposure. Despite their growing importance to public health, effective antiviral and preventive options remain limited for many emerging and reemerging vector-borne viruses due to viral genetic diversity, rapid evolutionary capacity, sporadic outbreak patterns, and economic constraints. While these viruses differ in taxonomy, genome organization, vector specificity, tissue tropism, and clinical manifestations, they exploit a shared vulnerability in host antiviral defense, particularly the timing of innate antiviral immunity to support viral replication, immune evasion, inflammatory dysregulation, and disease progression. Rather than simply suppressing antiviral defense, vector-borne viruses can delay early viral nucleic acid sensing, attenuate interferon induction or responsiveness, and extend the initial phase for viral replication. As viral burden increases and infected tissues undergo stress or damage, delayed immune activation can shift toward excessive inflammatory amplification, contributing to disease-specific pathology. In this study, we examine this temporal rewiring of innate antiviral immunity in representative vector-borne viruses such as dengue virus, chikungunya virus, and severe fever with thrombocytopenia syndrome virus and propose that understanding these conserved host dependencies may lead to broader, stage-specific, adaptable antiviral strategies that complement conventional virus-directed approaches.

## 1. Introduction

### 1.1. Emerging Vector-Borne Viral Infections as a Challenge for Virus-Specific Antiviral Development

Vector-borne viruses are transmitted to vertebrate hosts by hematophagous arthropod vectors such as mosquitoes and ticks. This occurs after the vectors acquire the virus from an infected host and replicate or persist within them [1]. These pathogens constitute diverse taxonomic groups, including flaviviruses, alphaviruses, and bandaviruses. Many of these viruses are zoonotic and can emerge sporadically at the human–animal–vector interface [2]. The global burden of vector-borne viral diseases has increased through the combined effects of climate change, altered precipitation patterns, land-use change, deforestation, urbanization, international travel, and global trade. These factors reshape vector survival, abundance, and geographic distribution, increase contact among vectors, reservoir hosts, domestic animals, and humans, and facilitate the spread of locally introduced viruses across regions [3,4,5,6,7]. As a result, pathogens that were historically restricted to specific ecological niches are now being recognized as regional or international public health threats.

Despite the growing burden of these diseases, developing virus-specific interventions remains challenging for many emerging and reemerging vector-borne viruses. Outbreaks are often sporadic, geographically limited, and clinically under-recognized, which limits case identification, complicates clinical trial design, and reduces incentives for continued investment in pathogen-specific therapeutics or vaccines [8,9]. Furthermore, most clinically significant vector-borne viruses are RNA viruses, and their error-prone replication process generates significant genetic diversity [10]. This evolutionary genetic plasticity can accelerate viral adaptation, the emergence of variants with altered fitness or vector competence, and the reduced durability of direct-acting antiviral strategies. Vector-borne viruses also differ in taxonomy, genome organization, replication strategy, vector specificity, tissue tropism, and disease phenotype. These biological differences make it difficult to develop broadly applicable antiviral agents when each pathogen is primarily approached through unique viral proteins or replication enzymes.

A further limitation of a purely virus-centered approach is that disease severity is determined not only by direct viral cytopathic effects but also by dysregulated host immune responses [11]. In many cases, excessive or aberrant immune activation contributes substantially to tissue damage and disease progression. Although vector-borne viruses belong to diverse taxonomic groups, many of them repeatedly manipulate host pathways that regulate nucleic-acid sensing, interferon signaling, and inflammatory amplification. The repeated exploitation of host biology suggests that shared host pathways may serve as conserved therapeutic entry points. Therefore, host-directed antiviral strategies could complement conventional, virus-specific approaches by targeting cellular processes necessary for viral replication, immune evasion, or pathogenesis. These interventions must be carefully designed according to the infection stage, cell type, and disease phenotype.

### 1.2. Host-Directed Antiviral Therapy as a Strategy Against Vector-Borne Viruses

Host-directed antiviral therapy is a therapeutic strategy that inhibits viral replication, persistence, or pathogenesis by modulating host cellular factors rather than directly targeting viral components. This approach fundamentally differs from conventional antiviral therapies. While conventional direct-acting antivirals target viral enzymes, structural proteins, or replication machinery, host-directed approaches aim to alter the cellular environment necessary for productive infection. These interventions can act through two broad mechanisms. First, they can restrict host processes that viruses need to enter, form a replication complex, replicate their genome, assemble, exit, and evade the immune system. Second, they may enhance antiviral defense or reduce host-mediated tissue injury when immune response becomes excessively activated and contributes to disease pathology [12,13]. Therefore, host-directed antiviral therapy is not only an alternative method of suppressing viral replication, but also a strategy that can modulate pathological host responses, thereby shaping clinical outcomes.

Based on this rationale, host-directed antiviral therapy is particularly relevant for vector-borne viral infections. Instead of focusing on highly variable viral targets, this approach focuses on recurrent host processes that are necessary for infection or pathogenesis. Rather than treating each virus as an entirely separate therapeutic problem, this approach aims to identify host dependencies repeatedly exploited by distinct viral systems that can be modulated within an acceptable safety window. Such targets may provide broader therapeutic benefits than inhibitors directed against a single viral protein. In addition, host genes are more genetically stable than viral genomes, making host-directed strategies less likely to cause classical resistance through mutation of a drug-binding viral target. However, viral adaptation through compensatory or alternative host-pathway utilization remains possible [12,14]. Host-directed antiviral therapy also offers practical advantages for outbreaks of infectious diseases. Many pharmacological agents that modulate inflammation and kinase signaling have been developed for non-infectious conditions. Repurposing these agents for antiviral applications can minimize the time, cost, and risk associated with developing new drugs, especially when their pharmacokinetic, pharmacodynamic, and safety profiles are already established in humans [15]. Furthermore, host-directed antiviral therapy can be effectively combined with direct-acting antivirals. In these strategies, the direct-acting component can reduce the amount of virus in the body, while the host-directed component may limit cellular processes that support replication or suppress inflammatory and tissue-damaging responses that cause severe disease [16].

Despite the attractive features of host-directed therapy, important challenges remain. Host pathways exploited by viruses are often essential for normal cellular physiology. Broadly inhibiting host pathways may compromise cell viability, tissue homeostasis, and protective immunity [16]. Furthermore, the function of a given host pathway can change throughout the course of an infection. For example, early interferon signaling may restrict viral replication, whereas delayed or prolonged inflammatory signaling may contribute to tissue injury [17]. Therefore, successful host-directed antiviral therapy requires more than identifying shared host dependencies. It requires the precise determination of the infection stage, the relevant cell type, the disease phenotype, and the therapeutic range within a given host pathway that can be safely and effectively modulated. Thus, the main challenge of host-directed antiviral therapy against vector-borne viruses is distinguishing host pathways that are only altered during infection from those that are necessary for viral replication or disease progression. A rational therapeutic approach should prioritize host pathways that are recurrently targeted by multiple viruses, pharmacologically modifiable, and distinct from essential physiological processes.

### 1.3. Shared Host-Pathway Dependencies Among Representative Vector-Borne Viruses

This study focuses on dengue virus (DENV), chikungunya virus (CHIKV), and severe fever with thrombocytopenia syndrome virus (SFTSV) as comparative models to examine host-pathway convergence across virologically distinct vector-borne viruses (Table 1). Although these viruses belong to distinct viral families, possess different evolutionary origins, are transmitted by different arthropod vectors, and exhibit distinct pathological manifestations, they repeatedly manipulate overlapping host processes including innate immune sensing, interferon signaling, and inflammatory networks (Table 2). Therefore, their comparison provides a useful framework for distinguishing virus-specific mechanisms from recurrent host dependencies that may be therapeutically targetable.

#### 1.3.1. Dengue Virus

**Figure 1:**
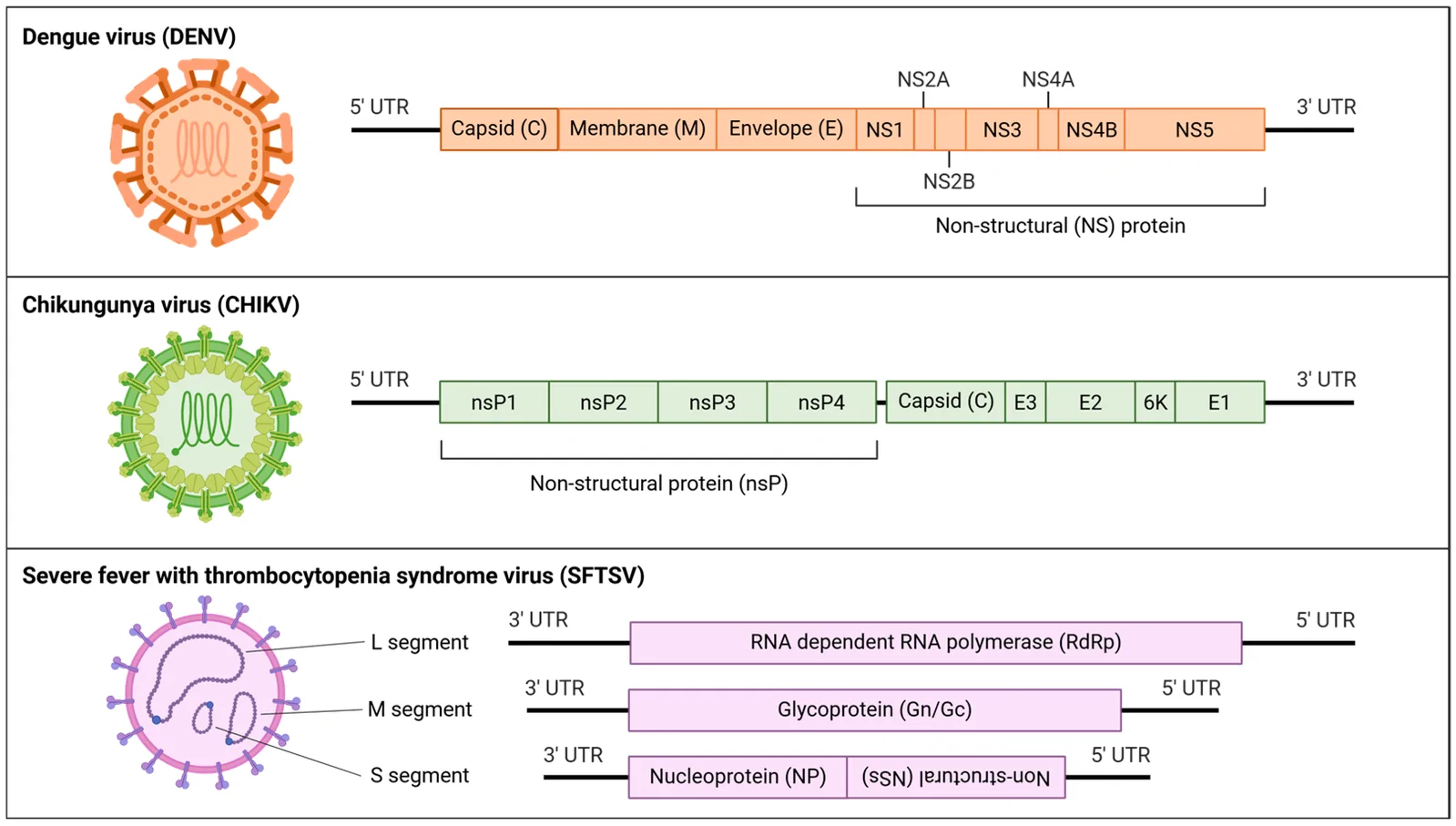
Genomic features of representative vector-borne viruses. Created in BioRender. Kim, E. (2026) https://BioRender.com/4vrv2gs (accessed on 24 June 2026).

Dengue virus (DENV), a member of the *Flaviviridae* family, is one of the most clinically important mosquito-borne enveloped positive-sense single-stranded RNA viruses and represents a major global public health burden [18]. The primary mosquito vectors responsible for viral transmission are *Aedes aegypti* and *Aedes albopictus*. Transmission occurs when infected mosquitoes inoculate virus-containing saliva into the skin during blood feeding. Following an incubation period of several days, DENV infection can cause an acute illness characterized by high fever, headache, myalgia, arthralgia, rash, and leukopenia [19]. In severe cases, infection may progress to dengue hemorrhagic fever (DHF) or dengue shock syndrome (DSS), both of which are associated with high morbidity and mortality. Notably, secondary infection with a heterologous serotype can result in antibody-dependent enhancement (ADE) and dysregulated hyperinflammatory responses, including cytokine storm, thereby significantly increasing disease severity and case fatality rates [18]. DENV possesses an approximately 11 kb RNA genome encoding three structural proteins—capsid (C), precursor membrane/membrane (prM/M), and envelope (E)—as well as seven non-structural proteins (NS1, NS2A, NS2B, NS3, NS4A, NS4B, and NS5) [20] (Figure 1).

#### 1.3.2. Chikungunya Virus

Chikungunya virus (CHIKV) is an enveloped, mosquito-borne positive-sense single-stranded RNA virus and a member of the family *Togaviridae*, genus *Alphavirus*. It has emerged as an important global cause of acute febrile illness and persistent inflammatory musculoskeletal disease [21]. Although CHIKV was previously associated primarily with geographically restricted outbreaks, recent decades have been marked by large epidemics across multiple continents, emphasizing its capacity for rapid geographic expansion and persistent public health consequences. Similar to DENV, the principal mosquito vectors responsible for CHIKV transmission are *Aedes aegypti* and *Aedes albopictus*, and viral transmission occurs when infected mosquitoes inoculate virus-containing saliva into the skin during blood feeding. Following an incubation period of approximately 3–7 days, a symptomatic infection typically presents with an acute onset of fever, severe polyarthralgia, myalgia, headache, rash, and, in some cases, gastrointestinal symptoms. A pronounced inflammatory response is a hallmark of acute CHIKV infection and contributes directly to pain, tissue inflammation, and disease severity. Intriguingly, a subset of patients subsequently develop chronic inflammatory arthropathy and persistent musculoskeletal symptoms that may last for months to years after the resolution of acute infection [22]. CHIKV possesses an approximately 12 kb RNA genome organized into two major open reading frames. The 5′ open reading frame encodes a non-structural polyprotein that is processed into four non-structural proteins, nsP1, nsP2, nsP3, and nsP4, which coordinate viral RNA capping, polyprotein processing, the formation of the replication complex, and genome replication. The 3′ open reading frame is expressed from a subgenomic RNA and encodes the structural polyprotein processed into the capsid (C), E3, E2, 6K, and E1 proteins [21] (Figure 1).

#### 1.3.3. Severe Fever with Thrombocytopenia Syndrome Virus

Severe fever with thrombocytopenia syndrome virus (SFTSV), also referred to taxonomically as *Dabie bandavirus*, is an enveloped tick-borne negative-sense single-stranded RNA virus belonging to the *Phenuiviridae* family. It is the etiological agent of severe fever with thrombocytopenia syndrome (SFTS), an emerging infectious disease associated with substantial morbidity and mortality [23]. Since its first identification in China in 2009, SFTS has been increasingly reported in East Asian countries, including China, South Korea, and Japan, raising growing public health concerns [24]. The principal arthropod vector responsible for viral transmission is the tick *Haemaphysalis longicornis*. In addition to tick-mediated transmission, human-to-human transmission has been reported through direct contact with blood or bodily fluids from infected individuals, particularly in healthcare or caregiving settings. Domestic animals and wild mammals may also participate in the ecology of SFTSV by serving as vertebrate hosts or amplifying reservoirs, thereby increasing opportunities for zoonotic spillover. Following an incubation period of approximately 5–14 days, infected individuals typically present with high fever, gastrointestinal symptoms such as nausea, vomiting, lymphadenopathy, thrombocytopenia, and leukopenia. In severe cases, infection can progress to systemic hyperinflammatory responses, coagulation abnormalities, neurological complications, multiple organ dysfunction (MOD), and death. Notably, SFTS is associated with a relatively high case fatality rate of approximately 30%, and no licensed vaccine is currently available; antiviral options remain limited and regionally variable [23]. Favipiravir has been approved in Japan for treating SFTS, but a globally established standard for antiviral therapy has yet to be developed [25]. SFTSV possesses a segmented RNA genome consisting of three segments designated L, M, and S. The L segment encodes the RNA-dependent RNA polymerase (RdRp), which is essential for viral replication and transcription, and the M segment encodes the envelope glycoproteins Gn and Gc involved in viral attachment and entry. The S segment encodes the nucleoprotein (NP) and non-structural protein (NSs) using an ambisense coding strategy [26] (Figure 1). Among these viral factors, NSs functions as a major virulence determinant and plays a critical role in antagonizing host antiviral immune responses.

Although DENV, CHIKV, and SFTSV differ substantially in viral taxonomy, genome organization, vector species, and clinical manifestations, these viruses exhibit convergence on common host pathways during infection. Despite these virological and pathological differences, all three viruses commonly target host pathways involved in antiviral immunity and inflammatory signaling. These shared host dependencies suggest that diverse vector-borne viruses utilize overlapping cellular mechanisms to support viral replication, evade immune restriction, and promote pathogenesis. In this review, “temporal rewiring” refers to virus-induced changes in the timing and coordination of host responses throughout the course of an infection. These changes can delay or attenuate early antiviral sensing and IFN-mediated restriction, which allows viral expansion before inflammatory responses become amplified and increasingly uncoupled from effective viral control. Therefore, identifying conserved host pathways that are functionally required for infection or pathogenesis provides a conceptual basis for host-directed antiviral strategies. The following sections examine these convergent pathways in greater detail, with particular emphasis on innate immune timing and their therapeutic implications.

## 2. Viral Rewiring of Innate Immune Timing

### 2.1. Antiviral Nucleic Acid Sensing and Interferon Signaling

Innate immune sensing constitutes the first line of host defense against virus infection. During vector-borne virus infection, viral nucleic acid intermediates including double-stranded RNA and uncapped or aberrantly structured viral RNA are detected by pattern recognition receptors (PRRs). These sensors include cytosolic RNA sensors such as retinoic acid-inducible gene I (RIG-I), melanoma differentiation-associated protein 5 (MDA5), and Toll-like receptors (TLRs). Activation of these PRRs initiates downstream signaling cascades that lead to the production of type I interferons (IFN-I) and, in some cellular contexts, type III interferons, followed by the induction of interferon-stimulated genes (ISGs) [27,28]. Collectively, these antiviral responses suppress viral replication, restrict viral dissemination, and contribute to the activation and coordination of adaptive immunity [29]. However, the IFN response should not be regarded as a simple linear antiviral pathway. Rather, it functions as a tightly regulated, time-dependent host defense program in which the timing, magnitude, duration, and cellular localization of signaling determine whether the response is protective or pathological [30,31,32]. Early IFN induction following infection generally limits viral replication before viral amplification occurs, thereby reducing viral burden and restricting systemic spread [30,31]. In contrast, delayed, prolonged, or dysregulated IFN and inflammatory signaling may fail to control viral replication efficiently while simultaneously promoting excessive cytokine production, immune dysregulation, tissue injury, and disease progression [32]. Thus, the biological outcome of IFN signaling depends not only on whether the pathway is activated but also on when and where it is activated during infection. Many vector-borne viruses take advantage of this temporal vulnerability. Successful viral infection does not necessarily depend on complete suppression of IFN signaling. Instead, viruses often employ strategies that transiently delay, attenuate, or compartmentalize antiviral sensing during the early phase of infection, thereby establishing a replication-permissive intracellular environment before robust antiviral responses are established [33]. As viral products accumulate and infected tissues are damaged, the host response may shift toward excessive inflammatory activation. This imbalance in timing links early immune evasion to later immunopathology, providing a mechanistic basis for understanding severe disease outcomes in vector-borne viral infections.

This temporal regulation of innate immunity is particularly relevant to DENV, CHIKV, and SFTSV. Although these viruses belong to distinct viral families and encode different immune antagonists, they converge functionally by attenuating early antiviral sensing and IFN-associated restriction, thereby extending a replication-permissive interval before inflammatory responses become a major determinant of pathology. Since the chronological boundaries of these states differ among viruses, experimental models, tissue compartments, and clinical reference points, the terms “early” and “late” are defined below.

### 2.2. Operational Definition of Early and Late Immune States Across Experimental and Clinical Settings

To clarify the temporal framework used throughout this review, it is important to distinguish three related but non-equivalent dimensions: timespan from experimental inoculation or symptom onset, underlying immunovirological state, and circadian phase. In this review, the terms “early” and “late” refer to operational immunovirological states rather than fixed chronological time periods. The early phase indicates the period when viral sensing and IFN/ISG induction can significantly restrict the initial rounds of viral replication before inflammatory circuits that damage tissue become dominant. The transition phase begins when antiviral control remains inadequate while viral loads and infected-cell stress accumulate, increasing PAMP- and DAMP-driven signaling, inflammatory cell recruitment, and cytokine amplification. The late phase refers to a state in which clinical pathology is increasingly sustained by dysregulated inflammation and tissue damage, even though viral replication may remain detectable. Therefore, the early-to-late transition is defined by a shift in the balance between effective antiviral restriction and immunopathology, rather than by a simple change from a “viral phase” to a “post-viral inflammatory phase”. These states vary based on the viral inoculum, cell type, tissue compartment, route of infection, host immune competence, and the reference point used to measure time. Table 3 shows this state-based framework in virus- and model-specific experimental and clinical windows. It also identifies candidate markers that help characterize transitions between immunovirological states.

Host circadian programs may be an additional source of temporal variation. Experimental studies have linked BMAL1/REV-ERB signaling to DENV replication and have demonstrated that REV-ERB pharmacological activation can suppress CHIKV replication and inflammatory gene expression [42,43,44,45]. However, these findings do not establish circadian phase-dependent disease progression or clinically validated times for changing from immune potentiation to inflammatory control, and comparable mechanistic evidence is currently lacking for SFTSV. Therefore, circadian phase is not incorporated into the operational definition of early and late infection states used in this review. However, sampling time and sleep–wake phase should be documented prospectively and incorporated as confounding variables in future longitudinal studies, especially when interpreting cytokine dynamics that may be affected by circadian variation.

### 2.3. Convergent Viral Strategies That Delay Early Interferon Induction and Signaling

**Figure 2:**
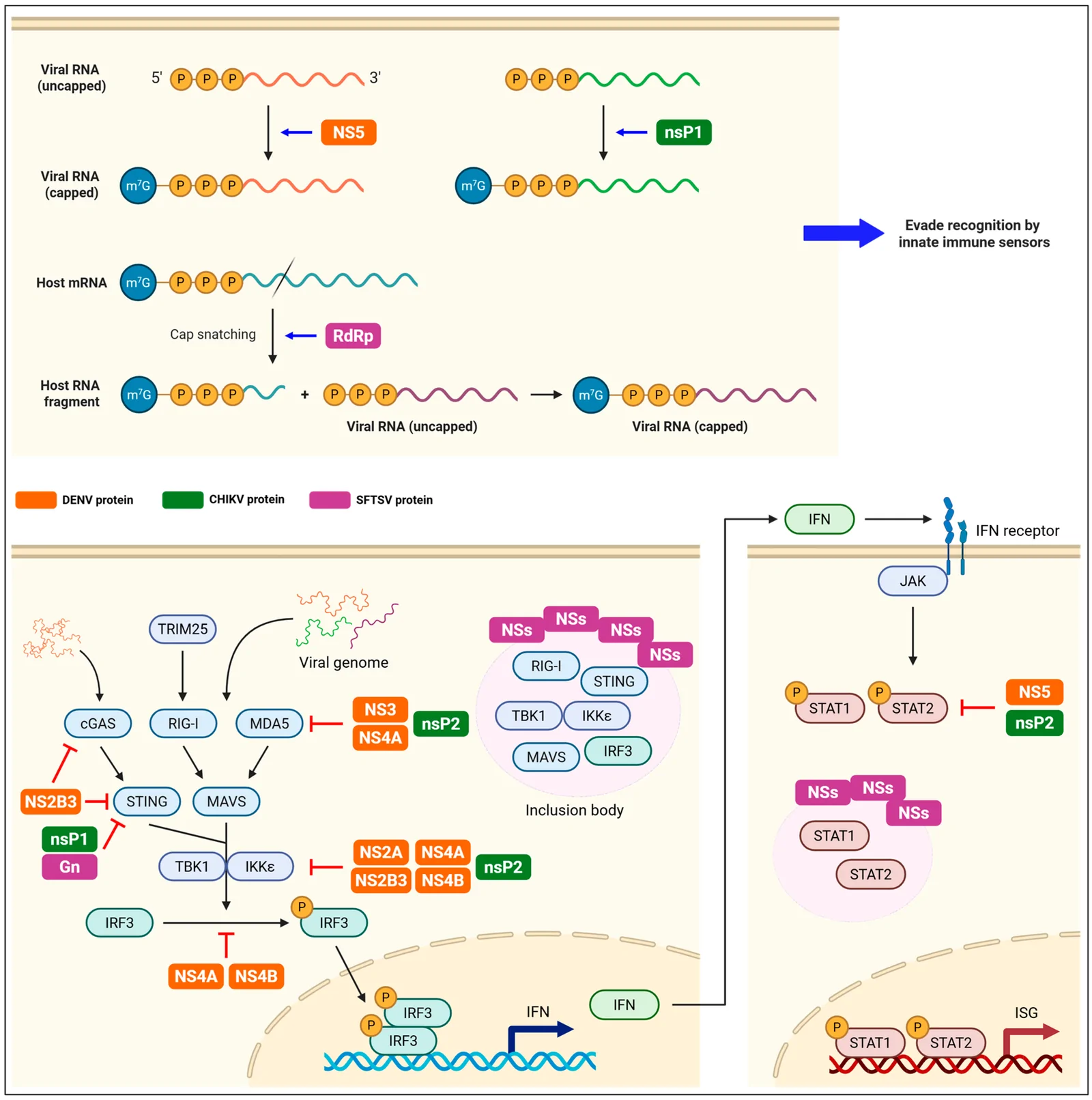
Convergent disruption of innate immune signaling pathways by vector-borne viruses. Created in BioRender. Kim, E. (2026) https://BioRender.com/zo2800z (accessed on 24 June 2026).

Many vector-borne viruses do not completely abolish innate immune signaling. Instead, they transiently delay, attenuate, or compartmentalize early antiviral responses, thereby extending the intracellular period during which viral replication can occur before a robust IFN-mediated antiviral state is established. Although DENV, CHIKV, and SFTSV differ significantly in genome organization and replication strategies, they converge functionally on multiple steps of the innate immune signaling pathway. These strategies include physically shielding viral replication intermediates, masking viral RNA with molecular mimicry, disrupting pattern-recognition receptor signaling, inhibiting the activation of downstream kinases and transcription factors, and suppressing JAK–STAT-dependent IFN responses (Figure 2).

The first shared strategy is the physical shielding of viral replication intermediates from innate immune sensors. DENV reorganizes the host ER membrane to generate vesicle-like replication compartments that physically sequester viral replication intermediates, including double-stranded RNA (dsRNA) from cytosolic sensors such as RIG-I and MDA5. As a result, viral dsRNA is largely concealed from cytosolic innate immune sensors [46,47]. CHIKV similarly remodels host cellular membranes to generate specialized replication structures known as spherules, which compartmentalize viral dsRNA and reduce its accessibility to cytosolic antiviral surveillance [48,49]. In contrast, SFTSV employs a distinct mechanism that does not primarily depend on membrane remodeling. SFTSV NSs protein forms viroplasm-like cytoplasmic inclusion bodies that sequester viral RNA and associated signaling molecules, thereby restricting access of host immune sensors to viral components [38,39,50,51,52].

The second convergent strategy is the molecular mimicking of viral RNA. DENV NS5 mediates 2′-O-methylation of the viral RNA cap structure through its methyltransferase activity, allowing viral RNA to mimic host mRNA and evade discrimination by innate immune sensors [53,54,55]. CHIKV nsP1 has both N7-methyltransferase and guanylyltransferase activities that are essential for formation of the viral 5′ cap structure and stabilization of viral RNA [56]. SFTSV, on the other hand, utilizes a cap-snatching mechanism in which the endonuclease domain of the viral RdRp cleaves capped host mRNA fragments and uses them to initiate viral transcription. This strategy allows SFTSV to acquire host-derived cap structures without the need for de novo cap synthesis, reducing immune recognition of viral RNA [57].

The third strategy is to directly disrupt PRR-adaptor antiviral signaling. Cytosolic RNA sensors such as RIG-I and MDA5 detect viral RNA and signal primarily through MAVS, whereas the cGAS-STING pathway, classically associated with DNA sensing, contributes to antiviral and inflammatory signaling during RNA virus infection by triggering cellular stress mechanisms [27,28,58]. DENV interferes with this signaling network at multiple points. DENV NS3 disrupts MAVS-associated signaling events required for efficient downstream antiviral activation whereas NS4A interferes with critical protein–protein interactions within antiviral signaling complexes [59,60,61]. The NS2B3 protease complex further suppresses innate immune signaling by targeting components of the cGAS-STING pathway, attenuating downstream IFN responses [62]. CHIKV suppresses RIG-I/MDA5-associated signaling through nsP2-mediated inhibition of antiviral transcriptional responses and interference with CARD domain-dependent signaling events [63]. CHIKV nsP1 has also been reported to interfere with STING-dependent signaling, thereby suppressing adaptor-mediated antiviral responses [64]. During SFTSV infection, NSs sequesters RIG-I- MAVS-, and STING-associated signaling components into cytoplasmic inclusion bodies, resulting in functional sequestration of these adaptor complexes [38,39]. Furthermore, SFTSV Gn glycoprotein directly antagonizes STING activation by preventing its dimerization and K27-linked ubiquitination, thereby inhibiting the serine/threonine kinases TANK-binding kinase 1 (TBK1) recruitment and Interferon Regulatory Factor 3 (IRF3) activation while promoting STING degradation [65].

Beyond interference with viral sensing and adaptor signaling, DENV, CHIKV, and SFTSV also target the downstream kinase-transcription factor cascade that links PRR adaptor activation to IFN responses. In this cascade, TBK1 and IkB kinase ε (IKKε) are activated downstream of adaptor proteins such as MAVS and STING and phosphorylate interferon regulatory factors IRF3 and IRF7, promoting their dimerization, nuclear translocation, and induction of IFN genes [27,28]. DENV NS2A and NS4B suppress TBK1/IKKε-associated signaling and inhibit IRF3 activation [60], whereas the NS2B3 protease complex further impairs kinase-dependent antiviral signaling [66]. CHIKV nsP2 similarly suppresses downstream antiviral transcriptional responses and limits IFN induction through interference with signaling pathways required for IRF3-dependent gene expression [63,67]. CHIKV infection further limits IRF3-dependent antiviral gene expression by inducing host translational shutoff, thereby preventing efficient IFN and ISG production [68]. SFTSV employs a distinct sequestration-based mechanism in which NSs directly binds TBK1 and IKKε and relocalizes them into cytoplasmic inclusion bodies, preventing downstream antiviral signaling [38,69]. SFTSV NSs also inhibits IRF3 phosphorylation, dimerization, and transcriptional activity through sequestration of signaling complexes and associated regulatory proteins [69]. Thus, although these three viruses have different viral proteins and molecular mechanisms, they all converge functionally on the suppression of IRF3-dependent IFN induction.

Once IFNs are produced, antiviral defense depends on JAK-STAT-mediated responses to IFNs. Activation of the IFN receptor stimulates JAK1 and TYK2, leading to the phosphorylation of STAT1 and STAT2, formation of the ISGF3 complex, nuclear translocation, and induction of ISGs [27,28,29]. In addition to limiting IFN induction, vector-borne viruses inhibit JAK-STAT signaling to blunt IFN responses. DENV NS5 targets STAT2 for proteasomal degradation, thereby suppressing downstream ISG expression [70]. CHIKV nsP2 inhibits STAT1 phosphorylation and nuclear translocation, preventing ISG expression [67]. SFTSV NSs suppresses IFN signaling through sequestration of STAT1 and STAT2 into cytoplasmic inclusion bodies [71,72]. These mechanisms demonstrate that DENV, CHIKV, and SFTSV not only suppress the production of IFNs upstream but also impair the ability of infected or neighboring cells to respond to IFN stimulation.

Taken together, DENV, CHIKV, and SFTSV interfere with innate antiviral immunity at distinct molecular entry points but produce a common systems-level outcome. By masking viral RNA, disrupting PRR-adaptor signaling, inhibiting TBK1/IKKε-IRF3 activation, and blocking JAK-STAT-dependent ISG induction, these viruses delay the formation of an effective antiviral state during the initial phase of infection. This delay allows viral replication to proceed before host restriction mechanisms are fully engaged. As viral products, infected-cell stress, and tissue damage accumulate, subsequent immune activation may become excessive and poorly coordinated, favoring cytokine amplification, inflammatory cell recruitment, and tissue injury. Therefore, early IFN antagonism by vector-borne viruses is not only an immune evasion mechanism but also a temporal remodeling strategy that links early viral amplification to later immunopathology.

### 2.4. From Interferon Delay to Inflammatory Pathology

In the early stages of infection, transient suppression of IFN responses facilitates efficient viral replication. However, the consequences of delayed antiviral signaling extend beyond increased viral replication [32,73]. Inadequate early restriction allows pathogen-associated molecular patterns (PAMPs) to accumulate with rising viral burden. At the same time, infected or injured cells release damage-associated molecular patterns (DAMPs) including cellular stress signals, endothelial mediators, complement activation products, and other inflammatory amplifiers [73,74,75,76,77]. As infection progresses, these signals drive excessive activation of innate immune pathways, shifting the host response from effective antiviral defense toward immunopathological inflammation. During this transition, persistent activation of innate immune pathways promotes the production of inflammatory cytokines and chemokines including TNF-α, IL-6, IL-1β, and CCL2 [73,75]. Although these mediators initially contribute to antiviral defense, immune cell recruitment, and tissue repair, sustained or spatially dysregulated inflammatory signaling can disrupt tissue homeostasis. Monocytes, macrophages, mast cells, endothelial cells, and tissue-resident immune cells can further amplify inflammation through positive feedback loops, resulting in vascular leakage, oxidative stress, coagulation abnormalities, tissue injury, and organ dysfunction [76,77]. Thus, severe disease reflects not simply the level of innate immune activation but the failure to coordinate timely inflammatory responses with antiviral restriction.

This temporal imbalance is particularly relevant to DENV, CHIKV, and SFTSV, whose early immune evasion is followed by inflammatory disease phenotypes that differ based on tissue tropism and host response mechanisms. In DENV infection, an initial NF-κB-dependent inflammatory wave can occur as early as 3–5.5 hpi, whereas a second wave at approximately 24 hpi coincides with productive replication, STAT1 activation, and inflammasome-associated cytokine release [34]. In patients, severity-associated cytokine patterns can diverge by day 3 of illness, before the clinical critical phase that typically occurs around the time of the fever peak on days 3–7 [19,35]. This interval is characterized not only by an increasing virus, but also by the accumulation of residual antigens, activated monocytes/macrophages and mast cells, complement and platelet responses, and endothelial injury. TNF-α, IL-6, MMP-2, and MMP-9 can weaken endothelial junction and basement membrane integrity, while NS1-associated endothelial signaling further promotes vascular permeability [78,79,80,81]. Therefore, a rational therapeutic change in dengue should be considered when the viral load is stable or declining through repeated testing, while the platelet count is decreasing, hemoconcentration is increasing, endothelial leakage is worsening, and inflammatory markers are rising.

In CHIKV infection, the first 48 h are particularly consequential. Experimental interruption of IFN-α signaling during this window increased acute dissemination and altered later persistence whereas later blockade had less effect [36]. The subsequent inflammatory phenotype is spatially concentrated in musculoskeletal tissues. Recent single-cell and spatial analyses identified CHIKV RNA in joint-associated inflammatory macrophages during chronic disease, together with increased NLRP3, IL-1b, antigen presentation programs, and adjacent IFN-γ-producing CD4+ T cells [82]. Fibroblast-rich synovial and periarticular niches provide a durable stromal scaffold for macrophage-T cell crosstalk, while CCL2-driven recruitment, IL-6/GM-CSF, COX-2-dependent prostaglandins, and osteoclastogenic pathways sustain pain, synovitis, and bone remodeling [83,84,85]. These anatomically retained cell populations explain why local joint inflammation can persist after systemic viremia has resolved and why tissue-directed or pathway-selective therapy may be preferable to broad systemic immune activation in the post-acute phase.

In SFTSV infection, early suppression of antiviral immunity allows SFTSV to replicate to high titers within the host. Serial clinical studies show that high viral burden, cytopenias, liver and tissue-injury enzymes, and inflammatory cytokines evolve together during the first 1~2 weeks. Viremia can persist for approximately 17 days and into the third week [40,41]. Mechanistically, SFTSV-induced mitochondrial DNA release activates the NLRP3 inflammasome while NSs-mediated NF-κB hyperactivation and impaired IFN signaling promote excessive IL-1β, IL-6, TNF-α, and IL-10 production [86,87,88,89]. Tissue localization also matters. Fatal human infections show SFTSV-positive plasmablast-lineage B cells and macrophages in secondary lymphoid and non-lymphoid organs, and humanized mouse models identify macrophage-rich spleen and liver as major target sites [90,91]. These niches may amplify acute systemic inflammation, cytopenias, and organ injury. In contrast to CHIKV, however, a well-established chronic tissue-resident inflammatory syndrome has not been demonstrated for SFTSV. Thus, current evidence supports tissue-compartmentalized amplification of acute disease rather than persistent post-acute inflammation.

## 3. Therapeutic Framework for Host-Directed Modulation of Innate Immune Timing

The pathological outcomes of vector-borne viral infections are strongly influenced by the temporal regulation of innate immune responses. As discussed above, insufficient antiviral signaling during the early stages of infection facilitates viral replication and dissemination whereas excessive and prolonged inflammatory activation during later stages can drive immunopathology and tissue injury. This temporal duality provides a mechanistic rationale for host-directed intervention. However, innate immune-targeted host-directed antiviral therapy should not simply aim to enhance or suppress IFN signaling. Instead, successful host-directed therapy requires stage-specific regulation of immune pathways according to viral kinetics, inflammatory status, tissue involvement, and disease phenotype. This principle is important because the same immune pathway can have protective or pathological effects, depending on the stage of the infection. In the early stages, strategies that enhance antiviral sensing and IFN responses can effectively restrict viral replication and dissemination. However, stimulating the same pathways during established inflammatory disease can worsen cytokine production, vascular injury, coagulation abnormalities, or tissue damage. Likewise, inflammatory control may be beneficial during late hyperinflammatory disease but detrimental if applied too early when antiviral restriction is still essential for controlling viruses. Therefore, the therapeutic objective should be precise temporal control of host responses within an appropriate therapeutic timeframe rather than maximal immune activation or suppression. But early and late stages of infection cannot be defined solely by days after symptom onset. Viral replication, antiviral immunity, inflammatory activation, and tissue injury do not necessarily progress in parallel. A patient at a later chronological time point may still have a high or increasing viral burden whereas inflammatory and tissue-damaging responses may emerge before viral clearance. Conversely, viral load may decline while vascular leakage, coagulation abnormalities, joint inflammation, or organ dysfunction continue to progress. Therefore, the decision to enhance antiviral immunity or suppress inflammation should not rely on a single chronological measure or molecular signature, but rather on an integrated assessment of viral kinetics, IFN and ISG responses, inflammatory activity, and tissue injury.

### 3.1. Early-Phase Immune Potentiation

Early-phase immune potentiation aims to counteract viral suppression of antiviral sensing and restore IFN-mediated antiviral defenses before extensive viral replication occurs. Because many vector-borne viruses transiently delay PRR signaling during early infection, therapeutic enhancement of innate immune activation during this early phase may suppress viral replication and prevent subsequent systemic dissemination [92]. Potential approaches include exogenous IFN administration, activation of PRR sensing through RIG-I, TLR or STING agonists, and innate immune priming strategies designed to establish a pre-activated antiviral state [12,13,93,94]. In DENV, peginterferon alfa-2a reduced viremia and accelerated viral clearance in rhesus macaques, whereas the RIG-I ligand 3p10LG9 transiently suppressed infection in human cell models; neither has demonstrated clinical efficacy [95,96]. The designed 5′-triposphate RNA ligand 3p10LG9 activates RIG-I and temporarily inhibits DENV infection in cell lines and primary human skin-derived immune cells. But it remains in the early preclinical stage. In CHIKV, topical imiquimod and the STING agonist cAIMP reduced viral burden and musculoskeletal pathology in preclinical models, but neither has been clinically evaluated [92,97]. In SFTSV, resiquimod reduced viral and inflammatory readouts in an IFNAR-blocked mouse model without improving survival, while retrospective data showed no significant clinical benefit of IFN-α [98]. Overall, these approaches remain largely preclinical, and their efficacy after systemic viral dissemination has begun remains uncertain (Table 4). The therapeutic efficacy of early innate immune potentiation is highly dependent on timing, dose, route of delivery, and disease context. Interventions that enhance PRR or IFN signaling before extensive viral replication may be protective, whereas the same interventions after inflammatory amplification has begun could intensify tissue injury. This concern is particularly relevant to DENV where excessive inflammatory signaling can contribute to endothelial dysfunction and vascular leakage and to CHIKV where persistent inflammatory activation is associated with arthralgia and chronic arthropathy. Similarly, it is relevant to SFTSV where late cytokine and NF-κB-associated inflammatory responses are linked to severe systemic disease. Therefore, early-phase immune potentiation should be considered a strategic approach with a narrow time range. It requires careful guidance based on the time from symptom onset, viral load, ISG signatures, cytokine profiles, and clinical indicators of tissue injury.

### 3.2. Late-Phase Inflammatory Modulation

During later stages of infection, the therapeutic priority may shift from antiviral potentiation to inflammatory control and disease tolerance. Since excessive inflammatory activation is a major cause of pathology, uncontrolled cytokine production and sustained immune cell activation contribute more substantially to tissue damage than direct viral replication itself. In this setting, host-directed strategies that restrict tissue-damaging inflammatory circuits can minimize immunopathology while maintaining sufficient antiviral defenses.

Potential approaches include transient modulation of JAK-STAT signaling, cytokine blockade, inflammasome-targeted therapy, complement modulation, preservation of endothelial barrier integrity, and pathway-specific inhibition of prostaglandin production or monocyte-recruitment programs [12,13,86,87,88,99]. The goal is not to suppress immunity entirely, but rather to interrupt the inflammatory response when it becomes excessive and impedes effective viral control. Representative pathology-directed HDTs are summarized in Table 5. In DENV, the IL-1 receptor antagonist anakinra has been evaluated in the Phase 2 AnaDen trial, although efficacy results remain unavailable [100], while baricitinib and dexamethasone are scheduled for evaluation in the Phase 3 DEN-HOST platform trial. The failure of early prednisolone to improve clinical or virological outcomes further suggests that broad, nonselective immunosuppression may not be sufficient [101]. In CHIKV infection, the NLRP3 inhibitor MCC950 and the MCP-family chemokine inhibitor bindarit reduced musculoskeletal inflammation and bone pathology in mouse models. However, MCC950 did not reduce viral replication, indicating that its principal effect was tissue protection rather than antiviral activity [102,103]. In SFTS, ruxolitinib and tocilizumab have shown preliminary clinical signals, but interpretation remains limited by nonrandomized comparisons, historical controls, corticosteroid co-treatment, and inconsistent findings across studies [104,105,106]. Overall, these candidates support selective targeting of cytokine, inflammasome, and monocyte-recruitment pathways, although none has yet been established as standard pathology-directed therapy. However, the timing of this approach must be carefully considered. Early inhibition of JAK-STAT signaling can impair ISG induction and compromise antiviral defense functions, potentially promoting viral replication. Similarly, late cytokine blockade or inflammasome inhibition may reduce inflammatory tissue damage but could also delay viral clearance, impair host defense, or increase susceptibility to secondary infection. Therefore, late-phase inflammatory control should be guided by biomarkers that distinguish ongoing viral replication from dominant inflammatory pathology. Furthermore, relevant parameters may include viral burden, interferon and ISG signatures, cytokine profiles, platelet counts, coagulation markers, endothelial injury markers, organ involvement, and tissue-specific inflammatory features.

### 3.3. Phenotype-Guided Therapeutic Prioritization

Therapeutic decisions should be guided by disease phenotype. DENV, CHIKV, and SFTSV do not cause the same pathological outcomes while converging on innate immune dysregulation. In severe dengue, vascular dysfunction, plasma leakage, thrombocytopenia, and shock are often the dominant clinical problems. Therefore, therapeutic strategies that preserve endothelial integrity, limit excessive cytokine or complement activation, and reduce vascular permeability may be particularly relevant during severe diseases [78,79,80,109]. In this context, uncontrolled immune activation during the inflammatory phase could potentially worsen vascular pathology.

CHIKV infection presents a different therapeutic problem. While acute viral replication contributes to the initial fever, the long-term disease burden is often caused by persistent musculoskeletal inflammation, activated macrophages, pain signaling, synovitis, and joint remodeling [83,84,85]. Persistent arthralgia after CHIKV infection has been associated with elevated IL-6 and GM-CSF, supporting the relevance of cytokine-driven inflammatory persistence in chronic disease [83]. Furthermore, COX-2-dependent prostaglandin production has been associated with post-chikungunya arthralgia, synovial inflammation, and bone destruction [85]. For this phenotype, therapies that target tissue-resident inflammatory networks such as those involving IL-6, GM-CSF, MCP-1/CCL2-mediated monocyte recruitment, COX-2-dependent prostaglandin production, and osteoclast-associated pathways, may be more effective than enhancing antiviral immunity systemically [83,84,85]. Therefore, CHIKV-associated chronic arthropathy illustrates that host-directed therapy must address post-acute inflammatory persistence rather than just acute viral suppression.

SFTSV is characterized by a systemic inflammatory phenotype involving thrombocytopenia, leukopenia, vascular injury, coagulation abnormalities, neurological complications, and, in severe cases, multiple organ dysfunction [86,87,88]. Severe SFTS has been associated with high viral load, abnormal cytokine profiles, and cytokine storm, including elevated IL-6, IL-10, MCP-1, and other inflammatory mediators [41,88]. In this context, therapeutic priorities may include controlling excessive cytokine and NF-κB-associated inflammatory pathways, preserving endothelial and coagulation homeostasis, and preventing progression to organ failure. However, severe SFTSV infection may also involve a high viral burden. Therefore, it is important to balance inflammatory restraint with the need to maintain residual antiviral immunity. These examples show that host-directed therapy cannot be chosen based on virus identity alone. It must be matched to the dominant pathological program present at a given stage of disease.

### 3.4. Drug Repurposing and Rational Combination Therapy

Drug repurposing is an attractive option for host-directed antiviral therapy against outbreaks of vector-borne viruses. Many agents that modulate interferon signaling, JAK–STAT pathways, cytokine activity, prostaglandin synthesis, complement activation, endothelial function, or inflammatory cell recruitment have already been developed for oncology, rheumatology, transplantation, metabolic disease, or inflammatory disorders [12,13,15]. Compared with de novo antiviral discovery, existing pharmacokinetic, pharmacodynamic, and safety data may shorten development timelines. This advantage is particularly significant for vector-borne viruses, as sporadic outbreaks, geographically restricted transmission, and limited commercial motivations often cause delays in conventional drug development [15,16]. However, repurposing should not be considered the same as immediate clinical availability. For example, a drug that is safe for chronic inflammatory diseases may pose different risks in acute viral infections, where early antiviral immunity is essential for controlling the virus [12,15,17]. Therefore, repurposed host-directed agents require infection-specific pharmacodynamic testing, careful dose selection, and defined therapeutic parameters based on biomarkers.

Combination therapy is more feasible than host-directed monotherapy. Where direct-acting antivirals are available, they can reduce viral burden, and host-directed agents may modulate the host pathways that regulate replication or pathology [12,13,15]. This complementary strategy may allow lower doses of individual agents, broaden therapeutic ranges, and reduce toxicity. For viruses without approved direct-acting antivirals, combinations of host-directed agents may still be considered but such approaches should be designed with non-overlapping toxicity, clear pathway engagement, and predetermined clinical stopping criteria.

## 4. Conclusions and Future Directions

Despite substantial differences in taxonomy, genome organization, transmission routes, and clinical manifestations, vector-borne viruses repeatedly target a limited number of host pathways. DENV, CHIKV, and SFTSV use distinct molecular mechanisms to delay early antiviral sensing, suppress IFN induction or responsiveness, and establish a replication-permissive environment during the initial phase of infection. As the viral load increases and the infected tissues become stressed or damaged, the immune response can shift toward excessive inflammatory amplification. This process connects early IFN antagonism with disease-specific immunopathology such as vascular leakage in severe dengue, persistent inflammatory arthropathy in chikungunya, and systemic hyperinflammation with multiorgan dysfunction in SFTS. This temporal framework has significant therapeutic implications. Host-directed antiviral therapy against vector-borne viruses should not be understood as either immunostimulation or immunosuppression. The same innate immune pathway can be protective in one phase of infection and pathological in another. Early restoration of viral sensing and ISG expression can restrict viral amplification before it spreads throughout the body. However, once inflammatory injury becomes dominant, therapeutic benefit may require the selective suppression of cytokine, endothelial, coagulation, and tissue-destructive programs while ensuring sufficient antiviral immunity. Therefore, the central challenge is not only to identify host pathways altered during infection but also to determine when, where, and in which disease phenotype those pathways can be safely and effectively modulated.

The future development of host-directed strategies will require therapeutic approaches guided by biomarkers. Biomarkers such as viral load, time from symptom onset, interferon and ISG signatures, cytokine profiles, platelet counts, coagulation parameters, endothelial injury markers, monocyte activation states, and tissue-specific inflammatory indicators may help distinguish patients for whom viral replication is the primary therapeutic target from those for whom immunopathology is the main driver of disease. These biomarkers may ultimately support selecting appropriate interventions, defining therapeutic timelines, monitoring pathway engagement, and establishing criteria for stopping immune-modulating therapies.

Therefore, future work should shift from static descriptions of virus–host interactions to temporal maps of host response states during infection. To identify the transition points when antiviral defense becomes uncoupled from inflammatory response, longitudinal analyses integrating viral kinetics, interferon activity, inflammatory amplification, vascular and coagulation changes, and tissue injury will be necessary. Furthermore, experimental systems should maintain the relevant immune and tissue contexts for host-directed therapy. Models that only maximize viral permissiveness may be inappropriate for evaluating interventions designed to modulate innate immunity, inflammatory control, or disease tolerance. Translation will require a more precise definition of actionable host states. Biomarkers should be developed for prognosis, treatment selection, pathway engagement, determining the therapeutic range, and safely discontinuing immune-modulating therapy. Repurposed immunomodulatory agents and combination strategies may offer practical advantages, particularly against highly contagious viruses, but they should be evaluated based on specific stages of the disease and disease phenotypes rather than being applied as broad-spectrum antivirals. A major unresolved task is distinguishing host pathways that are altered during infection from viral regulatory components that can be targeted without disrupting essential immune defenses or tissue homeostasis.

By placing immune timing at the center of therapeutic design, vector-borne viral diseases can be approached as dynamic host–pathogen processes rather than as problems of viral inhibition alone. This perspective does not replace virus-directed countermeasures but rather provides a complementary strategy for infections where viral diversity, sporadic emergence, and host-mediated pathology make conventional antiviral development difficult. A stage-resolved, phenotype-guided understanding of host responses could provide a more adaptable basis for antiviral intervention against current and future vector-borne viral threats.

## Figures and Tables

**Table 1:** Overview of representative vector-borne viruses.

Virus	Family	Genome	Vector	Clinical Manifestations	Innate Immune Pathology
DENV	*Flaviviridae*	+ssRNA	Aedes aegypti and Aedes albopictus mosquitoes	High fever, Hemorrhagic disease, Shock	Early IFN antagonism, monocyte/macrophage and endothelial activation, Vascular leakage and inflammatory amplification,
CHIKV	*Togaviridae*	+ssRNA	Aedes aegypti and Aedes albopictus mosquitoes	Acute/chronic arthritis	Innate immune evasion, Macrophage-associated inflammation, IL-6, GM-CSF, MCP-1/CCL2 and COX-2/PGE2-linked joint pathology
SFTSV	*Phenuiviridae*	−ssRNA (S segment ambisense)	Haemaphysalis longicornis ticks	Thrombocytopenia, Hemorrhagic complication, Multi organ dysfunction	NSs-mediated sequestration of IFN pathway components, Cytokine and NF-κB-associated inflammatory amplification, Endothelial and coagulation dysfunction

**Table 2:** Convergent innate immune pathways targeted by DENV, CHIKV, and SFTSV.

Host Pathway	Viral Proteins Involved			Functional Consequence
	DENV	CHIKV	SFTSV	
Viral genome sensor	NS2B3, NS3, NS4A, NS5	nsP1	RdRp, NSs	Impaired detection of viral nucleic acids
Viral RNA cap mimicry	NS5	nsP1	RdRp	Reduced discrimination of viral RNA from host RNA
PRR signaling adaptor	NS2B3 NS3, NS4A	nsP1, nsP2	NSs, Gn	Disrupted antiviral signal transduction
PRR signaling kinase and transcription factor	NS2A, NS2B3, NS4A, NS4B	nsP2	NSs	Impaired TBK1/IKKε-IRF signaling and IFN induction
JAK-STAT signaling	NS5	nsP2	NSs	Reduced ISG expression and antiviral activity

**Table 3:** Representative temporal windows and candidate markers of immunovirological state transitions.

Virus	Experimental Windows	Clinical Windows	Candidate Markers
**DENV**	3~5.5 hpi: first NF-κB-dependent inflammatory wave in human mononuclear phagocytes; approximately 24 hpi: a replication-associated second wave with combined NF-κB/STAT1 signaling and inflammasome-linked cytokine output [34].	Febrile/viremic phase, usually illness days 0~3/4; severity-associated cytokine divergence can be evident by day 3. Defervescence/critical phase generally occurs on days 3~7 [19,35].	Serial DENV RNA or NS1, defervescence, platelet and hematocrit trajectories, endothelial/vascular-leakage markers, and focused cytokines (e.g., IL-6, IL-8, IL-10, GM-CSF, CCL2).
**CHIKV**	0~48 hpi: IFN-α-sensitive window in mouse studies; acute musculoskeletal inflammation is commonly evaluated during approximately 3–7 dpi, whereas persistent disease is assessed from several weeks onward [21,36].	Acute viremic illness, approximately symptom days 0~7; subacute disease 1~12 weeks; chronic inflammatory musculoskeletal disease >12 weeks [37].	Plasma CHIKV RNA, fever resolution, CRP/ESR, IL-6/GM-CSF/CCL2, joint swelling and pain, and imaging or synovial inflammatory markers.
**SFTSV**	0~24 hpi: establishment and innate antagonism; 24~48 hpi: common mechanistic readout window for NSs-dependent signaling and cytokine responses, although model-specific thresholds vary [38,39].	High viremia, cytopenias, and cytokine dysregulation dominate the first 1~2 weeks; median viremia can persist for approximately 17 days and into week 3 [40,41].	Quantitative SFTSV RNA, platelet/leukocyte counts, AST/ALT, LDH, coagulation, IL-6/IL-10/IP-10, neurologic status, and evolving multiple-organ dysfunction.

Note: These windows are not treatment cutoffs. hpi, hours post infection; dpi, days post infection; CRP, C-reactive protein; ESR, erythrocyte sedimentation rate.

**Table 4:** Representative early-phase innate immune-potentiating interventions for DENV, CHIKV, and SFTSV.

Virus	Therapeutic Agent or Candidate	Host-Directed Strategy and Mechanism	Experimental Results	Development Stage	Ref.
**DENV**	Peginterferon alfa-2a (PEGASYS)	Exogenous type I IFN; restores IFN-mediated antiviral restriction	Reduced viremia and accelerated viral clearance in DENV-2-infected rhesus macaques; clinical benefit was not assessed.	Approved for other viral indications; DENV evidence remains limited to nonhuman-primate studies.	[95]
	3p10LG9	RIG-I agonist; induces IFN-dependent antiviral programs	Suppressed DENV infection in human cell lines and primary skin-derived immune cells; no in vivo data are available.	Experimental designer RNA at an early cell-based preclinical stage.	[96]
**CHIKV**	Imiquimod (Aldara 5% cream)	TLR7 agonist; induces a localized type I IFN and ISG response in skin-resident immune cells	Reduced CHIKV replication in mice and human skin explants; no clinical data are available.	Approved topical immunomodulator; CHIKV application remains preclinical.	[92]
	cAIMP	Synthetic cyclic dinucleotide STING agonist; activates STING-dependent IFN and ISG responses	Reduced viral burden and musculoskeletal inflammation in CHIKV-infected mice.	Experimental STING agonist at the mouse preclinical stage.	[97]
**SFTSV**	Resiquimod (R848)	TLR7/8 agonist (human), TLR7 agonist (mouse); promotes innate antiviral signaling	Reduced viral burden and inflammation in IFNAR-blocked mice without improving survival.	Early mouse proof of concept with no clinical or delayed-treatment data.	[98]

**Table 5:** Representative anti-inflammatory and tissue-protective host-directed interventions for DENV, CHIKV, and SFTSV.

Virus	Therapeutic Agent or Candidate	Host-Directed Strategy and Mechanism	Experimental Results	Development Stage	Ref.
**DENV**	Anakinra	IL-1 receptor blockade	Phase 2 AnaDen trial completed; efficacy results remain unpublished. An uncontrolled pediatric series reported 73% survival.	Phase 2 repurposing study; active, not recruiting, with efficacy results pending.	[100,101]
	Baricitinib and dexamethasone	JAK1/2 inhibition and broad cytokine suppression (respectively)	Planned for evaluation in the Phase 3 DEN-HOST trial; no efficacy data are available. Early prednisolone showed no clinical benefit.	Phase 3 platform trial not yet recruiting; planned initiation in October 2026.	[107,108]
**CHIKV**	MCC950 (CP-456,773)	Selective NLRP3 inflammasome inhibition	Reduced CHIKV-associated inflammation and bone loss in mice without lowering viral replication.	Experimental compound at the CHIKV preclinical stage.	[102]
	Bindarit	Inhibition of MCP-family chemokine synthesis and monocyte recruitment	Reduced joint swelling, monocyte recruitment, osteoclastogenesis, and bone loss in CHIKV-infected mice.	Preclinical; no efficacy studies in patients with CHIKV infection.	[103]
**SFTSV**	Ruxolitinib	JAK1/2 inhibition and attenuation of cytokine-associated hyperinflammation	A matched-control study suggested lower mortality and ICU admission, but the nonrandomized design limits interpretation.	Exploratory clinical proof of concept; randomized confirmatory trials are required.	[104]
	Tocilizumab	IL-6 receptor blockade	A single-center trial suggested lower mortality, although corticosteroid co-treatment confounded the effect; an observational study found no significant benefit over plasma exchange.	Promising repurposing candidate but not an established standard of care.	[105,106]

## Data Availability

No new data were created or analyzed in this study. Data sharing is not applicable to this article.
